# Two-Step 3D Microphone Array Fusion Algorithm to Enhance Sound Source Location Measurements

**DOI:** 10.3390/s26123798

**Published:** 2026-06-15

**Authors:** Mahya Shahmohammadimehrjardi, Bruce Wallace, Adrian D. C. Chan, Rafik Goubran, Pengcheng Xi

**Affiliations:** 1 Systems and Computer Engineering, Carleton University, Ottawa, ON K1S 5B6, Canada; adrianchan@cunet.carleton.ca (A.D.C.C.); goubran@sce.carleton.ca (R.G.); 2 SAM^3^ Innovation Hub, Ottawa, ON K1S 5B6, Canada; 3 Bruyère Health Research Institute, Ottawa, ON K1N 5C8, Canada; 4 National Research Council Canada, Ottawa, ON K1A 0R6, Canada; pengcheng.xi@nrc-cnrc.gc.ca

**Keywords:** microphone arrays, indoor 3D sound source localization, smart microphone subset choosing

## Abstract

This paper presents a novel two-step algorithm for microphone array fusion to enhance sound source localization (SSL) in three-dimensional indoor reverberant environments. Simulation analyses using simulated Room Impulse Responses (RIRs) reveal that Angle-of-Arrival (AoA) accuracy varies significantly with source position, causing certain microphone arrays to produce unreliable estimates. To mitigate this, the algorithm excludes microphone pairs with low-confidence AoAs, thereby improving overall localization accuracy. To extend the applicability of the approach, a generalized version of the algorithm is proposed for arbitrary room geometries and array positions on each wall. Its performance is assessed across three scenarios: (1) the original room geometry with arrays placed at the center of each wall; (2) a room with different dimensions; and (3) arrays placed at arbitrary positions on walls. The results show that the generalized algorithm achieves similar improvements as the original two-step method, approximately halving the localization error. Moreover, while room geometry and array placement influence SSL accuracy, the generalized method consistently reduces error across all cases. Three conventional AoA estimation methods are evaluated and their performance is compared in the baseline SSL. These findings highlight the robustness and practical value of the proposed algorithm on the baseline methods for improving SSL performance in acoustically challenging environments.

## 1. Introduction

Accurate three-dimensional sound source localization (SSL) in indoor environments remains a fundamental yet challenging problem in indoor environments. Practical deployments such as human–robot interaction, smart buildings, teleconferencing systems, and assistive technologies require reliable spatial awareness under reverberant and noisy conditions [1,2]. In enclosed spaces, multipath propagation, limited array aperture, and near-field acoustic effects introduce significant distortions in both temporal and spatial measurements, often leading to unstable or biased position estimates [3,4,5].

Conventional SSL approaches can be broadly classified into time-difference-based, beamforming-based, and subspace-based methods. Time-Difference-of-Arrival (TDoA) techniques estimate source position by intersecting hyperboloids derived from microphone pair delays [6]. TDoA values are commonly obtained using cross-correlation or Generalized Cross-Correlation with PHAse Transform (GCC-PHAT) [7], which enhances robustness to reverberation. While TDoA-based multilateration provides a direct geometric interpretation, small delay estimation errors propagate nonlinearly into large spatial errors, particularly when the sensor geometry is poorly conditioned [8].

Beamforming approaches, such as Steered Response Power with PHAT weighting (SRP-PHAT), evaluate candidate spatial locations by maximizing acoustic energy across the array aperture. These methods improve robustness in reverberant environments by integrating information across microphones and frequency bins, but they typically require dense spatial search grids and exhibit high computational complexity [9].

Subspace methods, including Multiple Signal Classification (MUSIC) [10], exploit the orthogonality between signal and noise subspaces to estimate the direction of arrival (DoA). Broadband extensions such as Incoherent Frequency-Bin MUSIC (IFB-MUSIC) integrate spectral information across multiple frequency bins to improve estimation stability [11]. Although subspace methods offer high angular resolution under ideal conditions, their performance in practical indoor environments is sensitive to model mismatch, limited array aperture, and reverberation [11]. In particular, standard MUSIC formulations assume far-field plane-wave propagation, which may introduce bias in near-field localization scenarios. There have been works that enhanced localization performance in outdoor far-field scenarios such as [12], where researchers focused on improving 3D drone localization accuracy by deploying two spatially separated microphone arrays and extending the GCC-PHAT-based TDOA framework to compute azimuth and elevation independently. By combining dual-array angle estimation with a hyperbolic intersection algorithm, they reduced angular bias that arises when AoA is approximated as azimuth in high-altitude scenarios. From a deep learning perspective, there are different methods that tried to increase SSL accuracy using DNN before. In [13], a simple DNN model is proposed that takes the normalized estimated AoAs from all microphone pairs as input and outputs the location of the sound source, which shows more accurate results compared to the incorporation of AoAs and triangulation for SSL. Also, ref. [14] proposed SpaceNet, a multimodal architecture that explicitly incorporates microphone geometry into the learning process. Instead of relying solely on acoustic features, their model decomposes spatial information into distances, azimuths, and elevations and fuses these with audio embeddings using modality-specific normalization.

Deep learning-based approaches have shown strong performance; however, they typically require large labeled datasets and extensive training procedures. For instance, supervised approaches rely on large-sized, physically realistic, precisely labeled datasets [15], and feature extraction pipelines can increase computational complexity [16].

To mitigate geometric instability, several studies have investigated microphone placement optimization and array topology design [17,18]. Increasing the number of microphones or optimizing array geometry can reduce the condition number of the localization problem and improve observability [19,20]. Probabilistic and maximum-likelihood frameworks have also been proposed to enhance robustness under diffuse acoustic fields [21].

Despite these advances, most existing methods implicitly assume that all available directional or delay measurements contribute equally to the final position estimate. In practical indoor environments, however, the reliability of directional information strongly depends on the relative spatial configuration between the source and each microphone array. Certain array–source geometries produce ambiguous or highly noisy DoA estimates due to reverberation, front–back ambiguity, or limited aperture. Fusion strategies that indiscriminately incorporate all available arrays may therefore amplify errors rather than reduce them. The accuracy of AoA estimation is inherently dependent on array geometry and directional sensitivity. In subspace-based methods such as MUSIC, the achievable resolution is governed by the conditioning and angular sensitivity of the array manifold, which can deteriorate under unfavorable geometric configurations [10]. For delay-based AoA estimation, the nonlinear sine relationship between inter-microphone time delay and incident angle further affects estimation stability. Since AoA is obtained through an inverse trigonometric transformation of the measured delay, small timing errors can be nonlinearly amplified, particularly in regions where the mapping exhibits increased sensitivity [6]. From a statistical standpoint, Cramér–Rao lower bound (CRLB) analyses show that AoA estimation variance depends on both source direction and array configuration, with certain geometries yielding inherently higher uncertainty [8]. These theoretical results indicate that AoA estimation performance is direction-dependent and can degrade under sharp or endfire incidence angles due to reduced geometric conditioning and nonlinear error propagation.

This observation motivates a shift from purely increasing sensor count or modifying estimation algorithms toward reliability-aware measurement selection. Rather than treating all arrays as equally informative, it is beneficial to evaluate the geometric consistency of each directional estimate prior to fusion.

This work proposes a two-stage three-dimensional SSL framework designed specifically for indoor near-field environments. In the first stage, conventional AoA-based processing is employed to obtain a coarse estimate of the source position. In the second stage, an adaptive omission mechanism evaluates the spatial consistency between estimated directions and the preliminary source location, selectively rejecting unreliable arrays before final position refinement. Unlike traditional approaches that rely primarily on denser arrays or more complex beamforming strategies, the proposed framework enhances localization accuracy through geometry-aware directional selection. Furthermore, a generalized formulation is introduced that operates independently of specific room layouts or predefined array configurations.

The main contributions of this paper are summarized as follows:

- A reliability-aware two-stage localization framework for indoor three-dimensional SSL.
- An adaptive array omission strategy based on spatial consistency constraints.
- A generalized formulation applicable to arbitrary array geometries.
- A comprehensive experimental comparison with classical TDoA multilateration, MUSIC, and broadband IFB-MUSIC baselines.

Extensive simulations conducted in reverberant indoor environments are used to evaluate the proposed framework. The results demonstrate improved localization accuracy compared to classical TDoA multilateration, MUSIC, and broadband IFB-MUSIC baselines. In addition to the proposed mapping-based hard selection, soft weighting strategies were also considered for comparison through cosine-based and Huber weighting functions. These approaches provide a gradual weighting of AoA contributions rather than strict inclusion or exclusion, enabling a comparison between hard omission and soft weighting schemes. These findings highlight the importance of reliability-aware directional selection in enhancing robustness under near-field and geometrically challenging conditions.

The proposed method is geometry-based and does not rely on training or extensive hyperparameter tuning, which facilitates reproducibility. The main parameters are directly related to the array configuration and angular selection criteria, and are explicitly defined in the corresponding sections. In contrast to data-driven approaches, the method is computationally lightweight and can be applied without prior training. In the broader context of signal interpretation and source localization, related studies in monitoring applications and acoustic emission localization highlight the importance of robustness under practical conditions and reproducibility across setups [22,23,24]. These works further motivate the development of geometry-based approaches that are less dependent on data-specific training and can generalize across environments.

## 2. System Model

In this paper, received signals at microphones are generated using Room Impulse Responses (RIRs) to study SSL methods, utilizing simulated RIRs from Pyroomacoustics library. A RIR describes how sound travels from a source point to a receiver point in a room, capturing the direct sound, reflections from surfaces, and reverberation effects [25].

To generate received signals at microphones, the RIR between each source location and each microphone is convolved with a raw audio signal (as the source signal) and an uncorrelated white noise is added to it later [26]. The received signal $s_{k}\left(t\right)$ at microphone *k* can be expressed as

(1) $$s_{k}\left(t\right)=h_{k}\left(t\right)*s_{\mathrm{raw}}\left(t\right)+w_{k}\left(t\right)\;$$

where

- $h_{k}\left(t\right)$: RIR between source location and microphone *k*;
- $s_{\mathrm{raw}}\left(t\right)$: Source signal (raw audio signal);
- $w_{k}\left(t\right)$: Uncorrelated white noise added to the received signal at microphone *k*;
- ∗: Convolution operation.

A 1-second Gaussian white noise signal is used as the raw signal to ensure uniform energy distribution across a wide frequency range. In practice, due to the finite sampling rate (48,000 Hz), the generated noise is effectively band-limited to 24,000 Hz, as dictated by the Nyquist theorem. Within this range, the amplitude spectrum remains approximately uniform. White Gaussian noise is used as a baseline input signal due to its broadband characteristics, which provide ideal conditions for time-delay estimation; however, additional experiments with speech signals are also considered to assess performance under realistic conditions.

### 2.1. RIR Simulation

**Figure 1:**
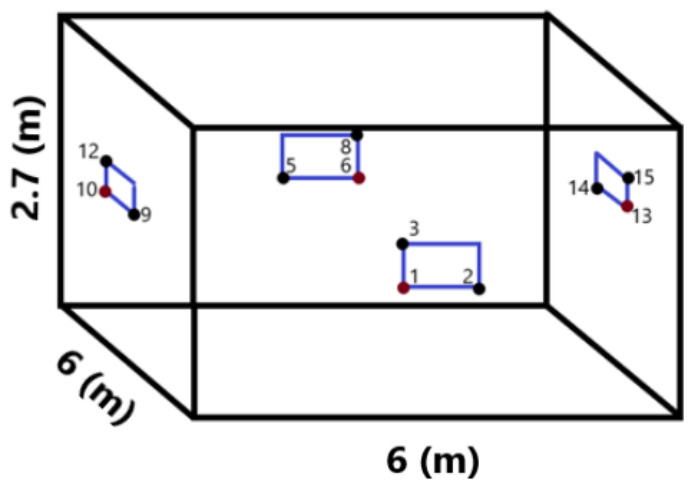
Colored dots represent the microphones selected from each array. The dark red dots indicate the reference microphones, while the two black dots in each array correspond to the microphones used for azimuth and elevation estimation.

The dimensions of UMIK-X-4 USB multichannel microphone array, comprising four microphone arrays, are used for simulation. UMIK-X-4 supports multichannel audio recording, allowing microphone arrays to be placed around the room, and future real evaluation to be possible. Each array consists of four microphones, with horizontal pairs separated by 0.16 m and vertical pairs by 0.13 m. As shown in Figure 1, three microphones from each array are utilized: one horizontal pair and one vertical microphone. The azimuth and elevation vectors are defined as described in Section 2.2.2 using horizontal and vertical microphone pairs respectively. Subsequently, the *x* and *y* coordinates of the source are estimated based on the azimuth vectors, using the *x* and *y* coordinates of the estimated point. Similarly, the *z* coordinate of the source is determined using the elevation vectors and the *z* coordinate of the estimated point. To enable flexible array selection, azimuth and elevation vectors are handled separately in the SSL process. This approach allows certain microphone pairs to be omitted when they do not contribute effectively. For example, for a specific source location (as shown later in this study), the vertical pair of an array improves localization accuracy, whereas the horizontal pair does not.

The source and microphone positions are set to generate RIRs. RIRs are calculated between each source position and each microphone. The raw audio signal is then convolved with these RIRs to simulate received signals at each microphone. To simulate realistic noise conditions, white Gaussian noise is added to each convolved signal according to a specified signal-to-noise ratio (SNR). Following the setup in [13], an SNR of 20 dB was used for all simulation models, except for the case shown in Section 3, which illustrates the SSL error distribution under different SNR levels. The same material properties as those used for the reverberant room in [13] were applied here as well, with an energy absorption coefficient of 0.6 and a scattering coefficient of 0.6. The resulting noisy, reverberant signals were saved as individual WAV files for subsequent analysis. All simulations were performed on a CPU (Intel i7-1065G7, 1.30 GHz, 8 GB RAM) without GPU acceleration.

### 2.2. Baseline SSL Method

This method serves as the baseline SSL approach in this paper, with the proposed methods for improved accuracy introduced in Section 2.3.1 and Section 2.3.2 as extensions of this foundation, and their results compared accordingly. The method is a 3D extension of the baseline SSL approach introduced in [13].

For completeness, the key elements of the method are briefly summarized here. The baseline approach estimates the source location by computing the AoA from multiple microphone pairs. Both azimuth and elevation angles are obtained from time-delay estimates between microphones. These AoAs are then represented as directional vectors originating from the centers of the corresponding microphone pairs. The source location is determined by combining these directional vectors using geometric relationships, where the intersection (or closest point) of the AoA vectors provides an estimate of the source position in three-dimensional space. This formulation enables direct localization without requiring prior training or data-driven models.

#### 2.2.1. Calculating TDoA and AoA

The estimation of the AoA is based on the TDoA between pairs of microphones. For each microphone pair, the TDoA is first estimated from the recorded signals, and then converted to an AoA using the known spacing between the microphones. Specifically, the time delay τ between two microphones separated by a distance *d* is related to the AoA θ through

(2) $$\theta =\arccos\left(\frac{c\;\tau }{d}\right),$$

where *c* denotes the speed of sound. The resulting AoA is then used to define a directional vector for source localization.

In this work, three TDoA estimation methods are considered and compared: Cross-Spectral Density (CPSD) with median-based phase unwrapping [13], peak of cross-correlation [13], and GCC_PHAT [7]. These methods differ in their robustness to noise and reverberation, which allows evaluation of the proposed SSL framework under varying signal conditions.

#### 2.2.2. Using Multiple AoAs for SSL

This process estimates the location of a sound source using multiple AoAs, as determining the distance with a single time delay is not feasible. The estimated source location is the point that minimizes the total distance to the lines representing possible source directions. Given multiple microphones, pairs are formed. For each pair the following steps are performed:

- The midpoint of the microphone pair is calculated as m.
- The AoA, θ, is estimated using any technique. Vertical pairs are used for elevation estimation where horizontal pairs are used for azimuth estimation. The AoA is represented by azimuth (az) and elevation (el).
- A vector perpendicular to each array towards the center of the room is used as the reference vector $u_{0}$, and then a proper rotation vector is applied to this vector to construct the unit direction vector u.
- The azimuth direction vector is rotated using the azimuth rotation matrix
  (3) $$R_{az}=\left[\begin{array}{ccc} \cos(-\mathrm{aoa}) & \sin(-\mathrm{aoa}) & 0\\ -\sin(-\mathrm{aoa}) & \cos(-\mathrm{aoa}) & 0\\ 0 & 0 & 1 \end{array}\right]$$
  Applying the rotation matrix:
  (4) $$u_{\mathrm{azimuth}}=R_{az}\cdot u_{0}$$
- A line using the direction vector and the midpoint of the microphone pair is constructed. The parametric line for azimuth is represented as
  (5) $$r_{\mathrm{azimuth}}\left(\alpha \right)=m+\alpha \cdot u_{\mathrm{azimuth}}$$
- Elevation Direction Vector with Rotation Matrix
  The elevation rotation matrix $R_{el}$ is determined by the alignment of the direction vector:
  - If the vector is aligned with the *y*-axis ([0,1,0] or [0,−1,0]), rotation is around the *x*-axis:
    (6) $$R_{el}=\left[\begin{array}{ccc} 1 & 0 & 0\\ 0 & \cos(-\mathrm{el}) & -\sin(-\mathrm{el})\\ 0 & \sin(-\mathrm{el}) & \cos(-\mathrm{el}) \end{array}\right]$$
  - But if the vector is aligned with the *x*-axis ([1,0,0] or [−1,0,0]), rotation is around the *y*-axis and $R_{el}$ is calculated as
    (7) $$R_{el}=\left[\begin{array}{ccc} \cos(\mathrm{el}) & 0 & \sin(\mathrm{el})\\ 0 & 1 & 0\\ -\sin(\mathrm{el}) & 0 & \cos(\mathrm{el}) \end{array}\right]$$
  The parametric line for elevation is represented as
  (8) $$r_{\mathrm{elevation}}\left(\beta \right)=m+\beta \cdot \left(R_{el}\cdot u_{0}\right)$$

#### 2.2.3. Calculating Perpendicular Distance to a Line

As explained, u is the unit direction vector corresponding to each microphone pair.

The candidate source location in space is $p={\left[x,y,z\right]}^{T}$, and the perpendicular distance to a line in space r(α)=m+α·u is given by

(9) $$\rho =\frac{∥C(u)\cdot (p-m)∥}{∥u∥}$$

where C(u) is the skew-symmetric matrixof u and is represented as

(10) $$C\left(u\right)=\left[\begin{array}{ccc} 0 & -u_{z} & u_{y}\\ u_{z} & 0 & -u_{x}\\ -u_{y} & u_{x} & 0 \end{array}\right]$$

Thus, the cross product becomes

(11) $$u\times \left(p-m\right)=C\left(u\right)\cdot \left[\begin{array}{c} x-m_{x}\\ y-m_{y}\\ z-m_{z} \end{array}\right]$$

#### 2.2.4. Optimization Used for Sound Source Location Estimation

To estimate the source location, the total distance from a candidate point to all azimuth lines is minimized, and the resulting *x* and *y* values are taken as the source’s *x* and *y* coordinates (*x* and *y* of the $p_{\mathrm{est}}$). A similar optimization is performed with elevation lines to determine the point with the minimum total distance, and the resulting *z* value is used as the source’s *z* coordinate (*z* of $p_{\mathrm{est}}$):

(12) $$p_{\mathrm{est}}=\arg\min_{p}\sum_{i}\rho_{i}\left(p\right),$$

where $\rho_{i}\left(p\right)$ is the distance to the *i*-th line.

While it is possible to apply both azimuth and elevation rotation matrices to a common reference vector (for each array) to construct a single unit direction vector, using the mean position of all microphones in each array as the midpoint to define one parametric line per array, this approach was not used. Instead, azimuth and elevation were treated separately in the SSL process to allow for selective elimination of microphone pairs in subsequent steps. For instance, horizontal pairs from one array can be excluded while retaining the vertical pairs. For example, horizontal pairs from an array can be excluded while retaining vertical pairs, allowing geometry-aware refinement of the localization process. Importantly, this staged formulation is mathematically consistent with the joint 3D approach and results in a negligible difference in localization accuracy (on the order of 0.001 m).

#### 2.2.5. Practical Interpretation

$p_{\mathrm{true}}$ denotes the known source position, and throughout this paper, the Mean Absolute Error (MAE) for *N* source positions is calculated as

(13) $$\mathrm{MAE}=\frac{1}{N}\sum_{n=1}^{N}\left|p_{\mathrm{true}}\left(n\right)-p_{\mathrm{est}}\left(n\right)\right|$$

Unlike Mean Squared Error (MSE), which squares the error and may disproportionately penalize large deviations, MAE provides a linear and more interpretable measure of localization error.

### 2.3. Proposed Methods for SSL

Simulation results obtained in [13] and further results discussed in Section 3.2 show that most errors in the baseline SSL method occur in the corners, with the underlying reasons explained thoroughly due to limitations in certain microphone pairs when estimating the correct AoA. To address this issue, the following methods are proposed as the extensions of the baseline SSL method.

#### 2.3.1. Two-Step SSL Algorithm

**Figure 2:**
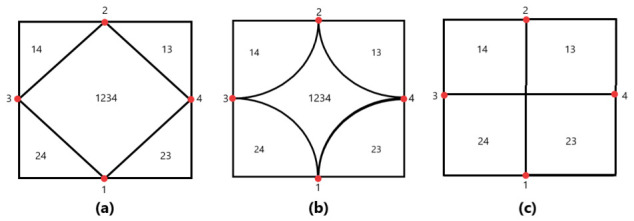
Three map plannings to minimize inaccuracies in azimuth estimation for SSL, addressing limitations of the microphone arrays for a 6×6×2.7 m room with microphone arrays placed at the center of each wall. (**a**) Corresponds to the planning directly derived from ±45° angular constraint. (**b**) is the modification of planning *a* expanding the boundaries towards the center and (**c**) is an aggressive extension of extending boundaries towards center. Red dots indicate the midpoints of horizontal microphone pairs within each array. The numbered labels represent the array identifiers, while the consecutive numbers within each section indicate the arrays used for azimuth estimation in those specific regions.

Figure 2 presents the mappings used for 3D SSL. The numbers illustrated in each subfigure indicate the horizontal microphone pairs used for azimuth estimation within each spatial region. These mappings are not defined heuristically; rather, the region boundaries are determined based on the angular alignment between the source-to-array direction vector and the normal direction of each microphone array. Specifically, only arrays whose viewing angle lies within the reliable operating range (approximately ±45°) are selected, ensuring stable AoA estimation.

Mapping shown in Figure 2a is directly derived from this ±45° angular constraint, forming regions where each array operates within its reliable range, as supported by the observations in Section 3 and prior studies [6,27,28]. Figure 2b extends this formulation by slightly expanding the region boundaries toward the center to improve robustness against initial localization errors, particularly for sources near region borders. Figure 2c represents a more aggressive extension of this idea; however, as shown in the results, it does not provide additional improvement, indicating that the primary benefit of boundary relaxation is already achieved in Figure 2b. For sources located in central regions with high confidence, using all microphone pairs remains preferable.

#### 2.3.2. Generalized Two-Step SSL Algorithm

The proposed two-step SSL algorithm is motivated by the observation that the accuracy of AoA estimation depends on the angular alignment between the source direction and the orientation of the microphone array. In particular, AoA estimates become less reliable when the source lies near endfire directions, where small errors in time-delay estimation can lead to large angular deviations. As a result, incorporating all available AoAs may introduce significant errors in the localization process.

To address this limitation, a geometry-aware refinement strategy is proposed, where only AoAs within a reliable angular range are considered.

The proposed two-step SSL algorithm for 3D localization has been further refined to operate independently of the room geometry and the specific placement of microphone arrays on the room walls.

In the generalized version, after obtaining an initial source estimate from the baseline SSL method, the algorithm evaluates the AoAs as vectors between the estimated point and the centers of all available microphone arrays. If all the obtained AoAs are within ±45°, all microphones are used for SSL; however, if even one of the estimated AoAs falls outside this range, only the two furthest microphone pairs are selected for SSL. The proposed selection strategy can be interpreted as rejecting AoA estimates with high uncertainty. While soft weighting approaches (e.g., cosine or Huber weighting) were also investigated, they were found to degrade performance, as highly inaccurate AoAs can still introduce significant bias even when down-weighted. In contrast, excluding such measurements prevents error propagation and results in more robust localization. The angular threshold therefore serves as a practical criterion to identify and discard unreliable AoA estimates.

For a specific scenario where the *x* and *y* dimensions of the room are the same and the microphone arrays are located at the wall centers, the generalized algorithm behaves the same as mapping shown in Figure 2a.

The selection of the ±45° threshold is based on empirical observations reported in [13], where it was shown that the reliable operating range for a microphone pair in the considered environment lies approximately within this interval.

## 3. Results

### 3.1. Comparing Three Basic Methods for AoA Estimation

**Figure 3:**
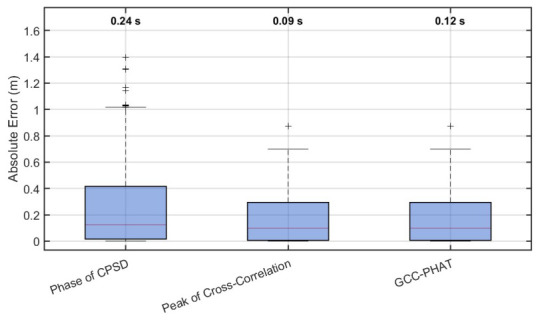
Absolute error distribution of three conventional methods for TDoA estimation across 729 equally spaced points in the 6×6×2.7 m room.

The error distribution for the baseline SSL method across 729 equally spaced source positions in the room was analyzed, using different AoA estimation methods: phase of the CPSD, peak of the cross-correlation, and GCC-PHAT. The absolute error distribution results are shown as boxplots along with execution time for each sound source shown in Figure 3.

The results shown in Figure 3 indicate that the error distribution values for all methods are very close to each other and as a result, the peak of cross-correlation method has been used in following evaluations for having less execution time between angle-based methods.

### 3.2. AoA Results for Baseline SSL

**Figure 4:**
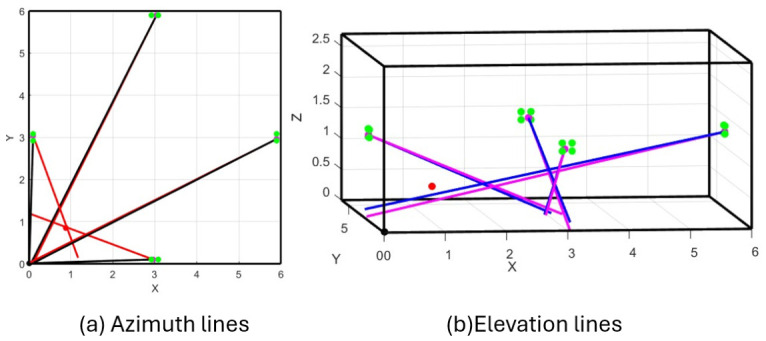
Green dots shows the microphone positions. The black dot shows the true source position and the red dot shows the estimated source position using baseline SSL method explained in Section 2.2. In (**a**), estimated azimuth lines are indicated by red lines where true azimuth lines are indicated by black lines. In (**b**), estimated elevation lines are indicated by blue lines and true elevation lines are indicated by purple lines.

**Figure 5:**
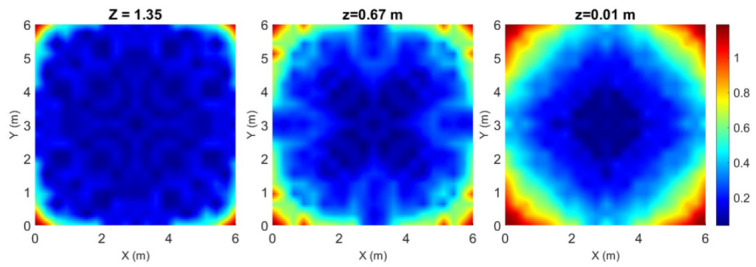
Absolute localization errors for 421 source positions at three heights in the 6×6×2.7 m room, visualized as a colormap. Dark blue and dark red represents lowest and highest absolute errors respectively.

The relationship between source distance and accuracy of estimated azimuth values is explained in [13] using real and simulated RIRs. Proposed mappings in [13] for increasing the 2D SSL accuracy has considered ±45° as the safe operation zone for microphone pairs for azimuth estimation. It is also derived from real RIR evaluation that the safe zone for azimuth estimation also decreases as the environment becomes more reverberant. As shown in Figure 4a and Figure 5, most errors occur in the corners. Figure 4 shows the estimated and true azimuth and elevation lines for a source position for one of the corners with maximum absolute error. It can be concluded from Figure 4 and Figure 7 that most inaccuracies arise from azimuth estimations using microphone arrays that have extreme azimuth angles to the source position.

**Figure 6:**
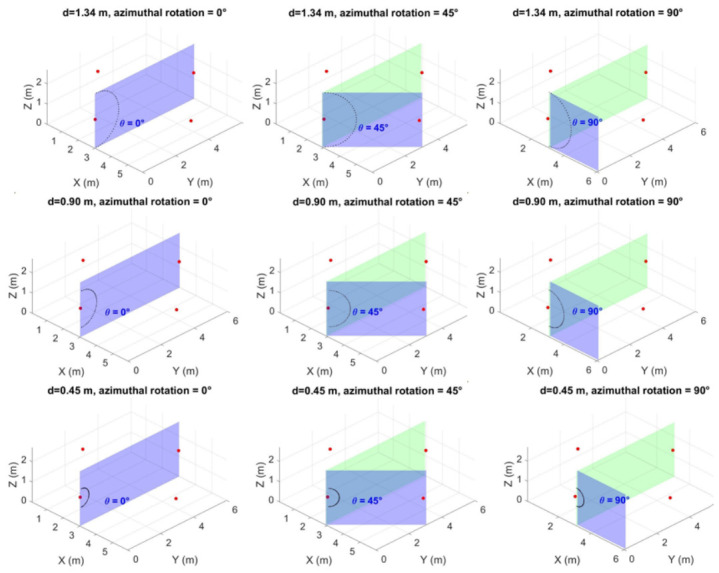
The purple plane represents the source plane containing the 37 source positions, equally distributed with an angular interval of $5^{\circ }$. The green plane represents the $0^{\circ }$ reference plane, parallel to the yz-plane. Red dots indicate the microphone array centers.

**Figure 7:**
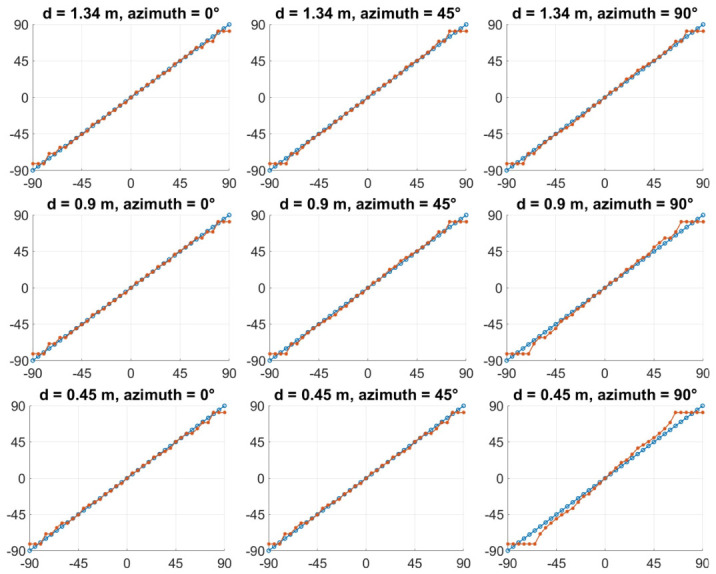
Elevation estimations for different sets of 37 sound source positions shown in Figure 6 using one vertical microphone pair for different distances and different azimuth values. Blue dots indicate true elevations and orange dots indicate estimated elevations. x and y axes show the true and estimated angles in degrees respectively.

To assess elevation estimation, 37 sound source positions were simulated at equal distances from the center of one microphone array, spaced by five-degree angular intervals, and lying on a plane perpendicular to the x–y plane. Three such planes at different distances from the array were considered to test the model across varying azimuth and distance combinations. Figure 6 illustrates the distribution of sound source positions on each plane, while Figure 7 presents the corresponding elevation estimation results. It is worth noting that treating azimuth and elevation jointly or separately in the baseline SSL yields similar localization accuracy. This indicates that the initial estimation stage is not sensitive to this design choice. Therefore, the separation is primarily motivated by the refinement stage, where azimuth estimates benefit from selective AoA usage while elevation estimates remain stable. Since elevation errors are negligible compared to azimuth errors across all cases, the mapping strategy is applied only to the selection of horizontal microphone pairs (azimuth), as shown in Figure 2, while all vertical microphone pairs (elevation) are retained for SSL.

### 3.3. Comparing Baseline SSL Results with Proposed Methods

**Figure 8:**
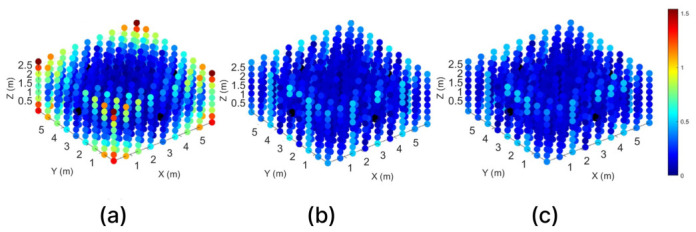
Colormap representation of absolute error for 729 sound source localizations using (**a**) baseline SSL method (Section 2.2), (**b**) two-step algorithm (mapping b) and (**c**) generalized two-step algorithm (Section 2.3.2) for the 6×6×2.7 m room.

**Figure 9:**
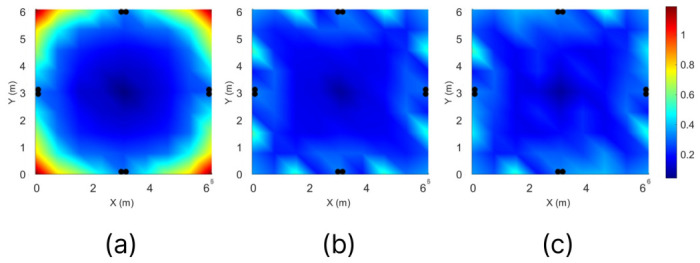
2D colormap of absolute localization errors for 729 sound source locations, averaged over source positions at the same height: (**a**) baseline SSL method (Section 2.2), (**b**) two-step algorithm (mapping b) and (**c**) generalized two-step algorithm (Section 2.3.2) for the 6×6×2.7 m room.

A total of 729 equally spaced source positions were generated within the room to evaluate the 3D SSL performance of the proposed methods in Section 2.3. Figure 8 shows the absolute localization errors of these 729 points, tested by the baseline SSL method described in Section 2.2, mapping.b from Figure 2 for the two-step algorithm, and the generalized two-step algorithm proposed in Section 2.3.2. Figure 9 represents 2D colormap representation of absolute localization errors, obtained by averaging the errors across all source positions at the same height. The comparison between the baseline SSL method and the proposed algorithms can be interpreted as an ablation study, where the second refinement step is removed. The observed performance improvement demonstrates the contribution of the refinement stage in enhancing localization accuracy by selectively incorporating reliable AoA estimates.

The baseline SSL method yielded a Mean Absolute Error (MAE) of 0.344 m, with the 25th and 75th percentiles of localization error at 0.110 m and 0.477 m, respectively, and a standard deviation of 0.309 m. When applying the proposed two-step SSL algorithm for 3D localization, as shown in Figure 2a, the MAE reduced to 0.188 m, with the 25th and 75th percentiles at 0.102 m and 0.250 m, and a standard deviation of 0.119 m. Further improvement was achieved using the mapping approach shown in Figure 2b, where the MAE decreased to 0.180 m, the 25th and 75th percentiles were 0.099 m and 0.247 m, and the standard deviation was 0.115 m. However, using the mapping configuration depicted in Figure 2c resulted in a slightly higher MAE of 0.183 m, with the 25th and 75th percentiles at 0.102 m and 0.247 m, and a standard deviation of 0.114 m. These results suggest that using more microphones in safe regions (center of the room) enhances localization accuracy.

By applying the generalized two-step SSL algorithm, which is adaptable to rooms with other dimensions and microphone placements that are not strictly centered on the walls, a MAE of 0.188 m is achieved for the 6×6×2.7 m room and arrays placed at wall centers. The 25th and 75th percentiles of the error are 0.103 m and 0.247 m, respectively, with a standard deviation of 0.118 m. These results demonstrate that the generalized algorithm works such as mapping depicted in Figure 2a as expected.

To assess statistical significance, 30 independent estimates were generated for each of the 125 equally spaced source locations for both the baseline and proposed methods. A paired *t*-test was conducted on the resulting localization errors. The proposed method reduces the mean localization error by 0.145 m compared to the baseline, and this improvement is statistically significant (p<0.001). The variability across trials is reflected by a standard deviation of 0.235 m. The 95% confidence interval for the mean error reduction is [X, Y] m. The corresponding effect size (Cohen’s d=0.619) indicates a moderate-to-large practical improvement, supporting the effectiveness of the proposed refinement strategy.

**Figure 10:**
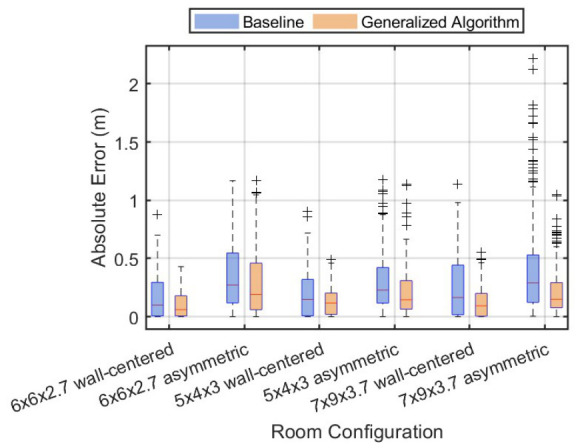
Error distribution results for three different room geometries and two different microphone array positions using baseline and generalized algorithm.

For further evaluation, the generalized algorithm was applied to different room dimensions and non-symmetric array positioning with the same materials used before (energy absorption coefficient of 0.6 and a scattering coefficient of 0.6 and SNR 20 dB). Figure 10 demostrates absolute error distribution results across 729 source positions for different room dimensions: 6×6×2.7 m, 5×4×3 m and 7×9×3.7 m. The array center positions for asymmetric scenarios are shown in Table 1.

**Figure 11:**
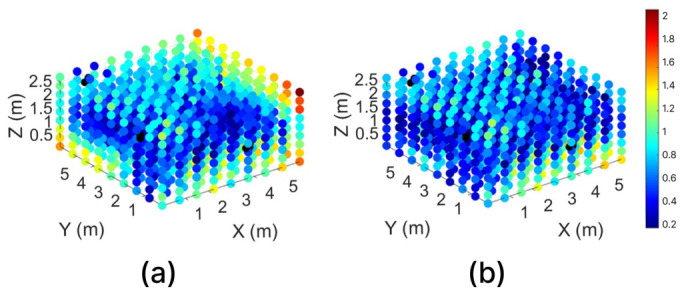
Colormap representation of absolute error for 729 equally spaced sound source locations using (**a**) baseline SSL method (Section 2.2), (**b**) generalized two-step algorithm (Section 2.3.2) for asymmetric microphone array positions for the 6×6×2.7 m room.

The results shown in Figure 10 show that absolute error reduceds significantly after applying the generalized two-step algorithm proposed. Although asymmetric placement can reduce coverage blind spots, it degrades the conditioning of the measurement geometry. In this case, random array placement results in uneven and poorly intersecting angular constraints, increasing geometric dilution of precision and amplifying the effect of angular estimation errors, which outweighs the benefits of improved coverage and leads to higher absolute localization error [29]. Figure 11 shows a colormap representation of the absolute localization error for an asymmetric array configuration. Although the generalized algorithm reduces the error, the overall error remains higher than that observed for the symmetric array configuration.

These results also indicate that, when the position of the arrays is randomly chosen, absolute errors using the baseline SSL increases compared to the scenario where the arrays are positioned at the center of each wall; however, generalized mapping still decreases the errors significantly.

To evaluate the performance under more realistic conditions, additional experiments were conducted using speech signals with different spectral characteristics. Compared to white Gaussian noise, a decrease in localization accuracy was observed. This is attributed to the structured and non-stationary nature of speech signals, which introduces ambiguity in cross-correlation-based time-delay estimation, resulting in less distinct and sometimes multiple correlation peaks. Since the proposed mapping strategy relies on the initial AoA estimation to select appropriate microphone pairs, inaccuracies in delay estimation can lead to incorrect region selection and consequently reduced localization accuracy. In contrast, white Gaussian noise provides a broadband excitation that yields sharper and more stable correlation peaks, facilitating more reliable AoA estimation. These results indicate that the white noise assumption introduces an optimistic bias in performance evaluation. The findings highlight that the effectiveness of the proposed mapping strategy depends on the reliability of the underlying AoA estimation, and improving robustness for real-world signals remains an important direction for future work.

### 3.4. Comparison: Other Methods to Mitigate AoA Inaccuracies

There are other approaches that try to mitigate the error rising from some AoAs. The solutions proposed in these papers are different types of weighting. Here, two of these methods are applied and compared to the proposed two-step algorithm.

Cosine weighting: The first weighting strategy is geometry-based, inspired by prior works that assign weights to AoA lines depending on their orientation or informativeness [4,30]. The MAE on 729 sound sources using cosine weighting is 0.270 m while the MAE for the proposed algorithm is 0.181 m.

**Figure 12:**
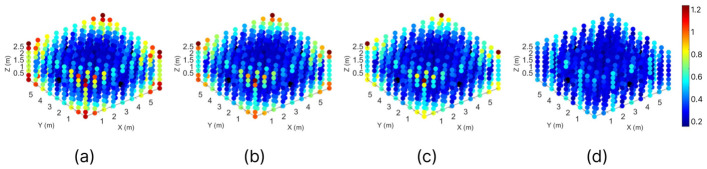
Colormap representation of absolute error for 729 equally spaced sound source locations using (**a**) baseline SSL, (**b**) cosine weighting, (**c**) cosine weighting combined with Huber weighting and (**d**) proposed algorithm for the 6×6×2.7 m room.

Huber weighting: The second weighting strategy is residual-based, using Huber’s M-estimator [31] to reduce the impact of inconsistent AoAs. Robust weighting has been studied in bearing-only localization [32]. Figure 12 shows the comparison result between applying only cosine weighting, applying cosine + Huber weighting on AoAs and the proposed algorithm where some AoAs are discarded. The MAE on 729 sound sources using hybrid weighting is 0.250 m and, while it is higher than proposed algorithm by 0.069 m, it is lower than only using cosine weighting by 0.020 m.

Other state-of-the-art methods used for comparison include MUSIC [10,33] and IFB-MUSIC [11]. The results indicate that both approaches yield a MAE of approximately 2 m. Although these techniques are widely recognized for their high accuracy under narrowband conditions, their performance degrades when applied to wideband signals. In this scenario, where white noise is used as the sound source, the broadband nature of the signal further exposes the inherent limitations of these methods, leading to reduced localization accuracy.

### 3.5. Comparison: Comparing Baseline Method and Generalized Algorithm in Different Frequencies and SNRs

**Figure 13:**
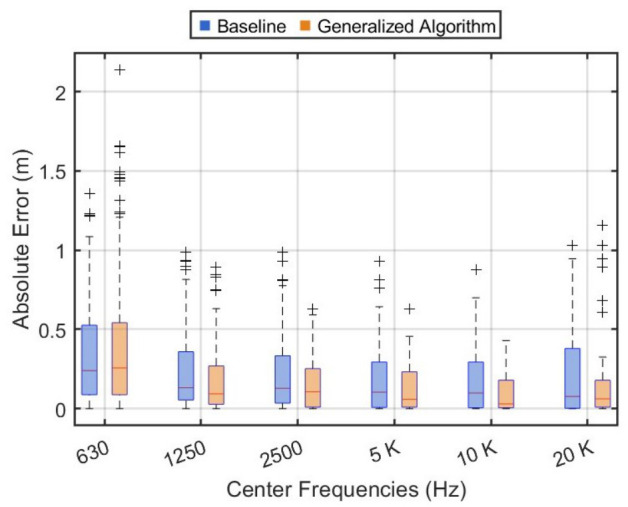
Error distribution results for baseline and generalized algorithm at different frequency bands shown in boxplots.

**Figure 14:**
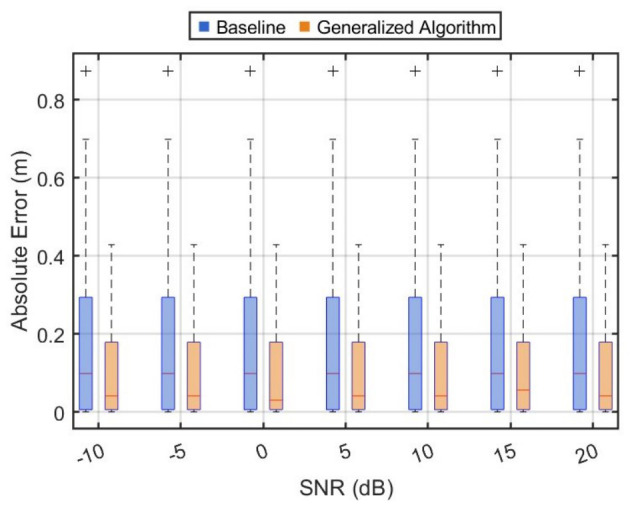
Error distribution results for baseline and generalized algorithm with different SNR levels for the 6×6×2.7 m room shown in boxplots.

Here, the baseline method and proposed generalized algorithm have been compared in the 6×6×2.7 m room for different scenarios: (1) sound source in an environment with different noise levels (SNR ranging from −10 dB to 20 dB), and (2) sound source with different frequency bands; the white noise sound signal was filtered into octave frequency bands, where the upper cutoff frequency is twice the lower cutoff frequency. Figure 13 illustrates that the generalized algorithm has lower overall error except for the frequency band with 630 Hz center frequency. Figure 14 shows that both methods are robust to noise.

## 4. Conclusions

It can be concluded that when an AoA-based method is used and the microphone arrays are placed at the center of the walls, the MAE can be reduced by 50% through a straightforward yet carefully designed mapping approach, without relying on complex or computationally expensive methods such as deep learning techniques [13].

The results presented in this study confirm that both the proposed two-step method and its generalized variant effectively reduce localization error across diverse configurations, including multiple room dimensions and asymmetric array placements. These findings highlight the robustness and adaptability of the proposed approach, showing that optimizing array selection based on spatial and angular relationships between arrays and the estimated source position is a key factor in improving AoA-based SSL accuracy.

Furthermore, the generalized algorithm eliminates the dependency on specific room geometries or microphone placements, making it applicable to a broader range of acoustic environments. The consistency of improvement across test conditions suggests that the proposed mapping and array selection mechanisms can serve as a practical framework for real-world deployments of indoor localization systems.

Future work will focus on extending this framework to more complex conditions, including rooms with variable surface absorption characteristics and irregular geometries, dynamic array configurations, and moving sources, as well as outdoor environments with higher noise and wind interference. While the proposed method is validated through simulations under controlled conditions, real-world implementation may introduce additional challenges that can affect time-delay estimation and, consequently, the reliability of AoA-based mapping. A comprehensive real-world experimental validation is part of ongoing work and will be addressed in future studies. Future work will also focus on improving robustness to realistic signals such as speech by incorporating more reliable delay estimation and confidence-based array selection strategies. Additionally, integrating near-field modeling and hybrid AoA–TDoA fusion techniques may further enhance localization precision and stability under reverberant conditions.

## Figures and Tables

**Table 1:** Asymmetric microphone array positions for different room dimensions.

Array Position	6 × 6 × 2.7 m	5 × 4 × 3 m	7 × 9 × 3.7 m
Front wall	(2, 0.1, 1)	(2, 0.1, 1)	(4, 0.1, 1)
Back wall	(4, 5.9, 2)	(3, 3.9, 2)	(3, 8.9, 3)
Left wall	(0.1, 2.1, 0.8)	(0.1, 1.5, 0.8)	(0.1, 4, 1.5)
Right wall	(5.9, 3.8, 2.1)	(4.9, 2.5, 2.1)	(6.9, 5, 2.5)

## Data Availability

The raw data supporting the conclusions of this article will be made available by the authors on request.
